# (*E*)-4-Meth­oxy-*N*′-[(pyridin-4-yl)methyl­idene]benzohydrazide monohydrate

**DOI:** 10.1107/S1600536812034988

**Published:** 2012-08-25

**Authors:** Muhammad Taha, Humera Naz, Aqilah Abd Rahman, Nor Hadiani Ismail, Yousuf Sammer

**Affiliations:** a Atta-ur-Rahman Research Institute for Natural Products Discovery (RiND), Universiti Tecknologi MARA, Puncak Alam, 42300 Selangor, Malaysia; b Faculty of Pharmacy, Universiti Tecknologi MARA, Puncak Alam, 42300 Selangor, Malaysia; c H.E.J. Research Institute of Chemistry, International Center for Chemical and Biological Sciences, University of Karachi, Karachi 75270, Pakistan

## Abstract

In the title compound, C_14_H_13_N_3_O_2_·H_2_O, the azomethine double bond adopts an *E* conformation and the N—N=C—C torsion angle is 178.37 (19)°. The dihedral angle between the benzene and pyridine rings is 5.58 (12)° and the C atom of the meth­oxy group is roughly coplanar with its attached ring [deviation = 0.157 (3) Å]. In the crystal, the components are linked by O—H⋯O, O—H⋯N, N—H⋯O and C—H⋯O hydrogen bonds, forming (001) sheets. The water O atom accepts one N—H⋯O and two C—H⋯O inter­actions from the adjacent organic mol­ecule.

## Related literature

For the biological activity of benzohydraazides, see: Bayrak *et al.* (2009 ▶). For the crystal structures of related benzohydrazides, see: Taha *et al.* (2012) ▶; Fun *et al.* (2011 ▶); Lu *et al.* (2009 ▶); Zhang (2009*a* ▶,*b* ▶).
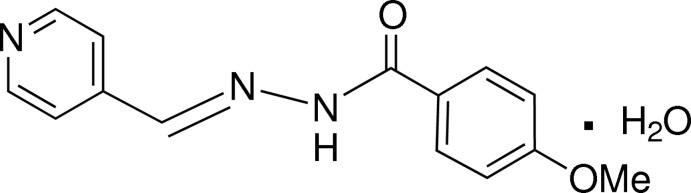


## Experimental

### 

#### Crystal data

C_14_H_13_N_3_O_2_·H_2_O
*M* *_r_* = 273.29
Monoclinic, 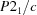

*a* = 6.6878 (5) Å
*b* = 7.0420 (5) Å
*c* = 29.249 (2) Å
β = 94.233 (2)°
*V* = 1373.74 (17) Å^3^
*Z* = 4
Mo *K*α radiation
μ = 0.10 mm^−1^
*T* = 273 K
0.20 × 0.17 × 0.10 mm

#### Data collection

Bruker SMART APEX CCD diffractometer
Absorption correction: multi-scan (*SADABS*; Bruker, 2000 ▶) *T*_min_ = 0.981, *T*_max_ = 0.991
7767 measured reflections
2560 independent reflections
1548 reflections with *I* > 2σ(*I*)
*R*_int_ = 0.038

#### Refinement

*R*[*F* ^2^ > 2σ(*F* ^2^)] = 0.047
*wR*(*F* ^2^) = 0.146
*S* = 1.03
2560 reflections
182 parameters
H-atom parameters constrained
Δρ_max_ = 0.16 e Å^−3^
Δρ_min_ = −0.20 e Å^−3^

### 

Data collection: *SMART* (Bruker, 2000 ▶); cell refinement: *SAINT* (Bruker, 2000 ▶); data reduction: *SAINT*; program(s) used to solve structure: *SHELXS97* (Sheldrick, 2008 ▶); program(s) used to refine structure: *SHELXL97* (Sheldrick, 2008 ▶); molecular graphics: *SHELXTL* (Sheldrick, 2008 ▶); software used to prepare material for publication: *SHELXTL*, *PARST* (Nardelli, 1995 ▶) and *PLATON* (Spek, 2009 ▶).

## Supplementary Material

Crystal structure: contains datablock(s) global, I. DOI: 10.1107/S1600536812034988/hb6930sup1.cif

Structure factors: contains datablock(s) I. DOI: 10.1107/S1600536812034988/hb6930Isup2.hkl

Supplementary material file. DOI: 10.1107/S1600536812034988/hb6930Isup3.cml

Additional supplementary materials:  crystallographic information; 3D view; checkCIF report

## Figures and Tables

**Table 1:** Hydrogen-bond geometry (Å, °)

*D*—H⋯*A*	*D*—H	H⋯*A*	*D*⋯*A*	*D*—H⋯*A*
O1*W*—H1⋯O1^i^	0.84	2.00	2.811 (2)	162
O1*W*—H2⋯N3^ii^	0.91	2.11	2.956 (3)	154
N1—H1*A*⋯O1*W*	0.86	2.08	2.911 (2)	161
C1—H1*B*⋯O1*W*	0.93	2.54	3.440 (3)	162
C8—H8*A*⋯O1*W*	0.93	2.48	3.272 (3)	143
C11—H11*A*⋯O2^iii^	0.93	2.47	3.375 (3)	165

Symmetry codes: (i) 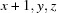; (ii) 
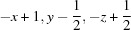
; (iii) 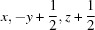.

**Fig. 1.**
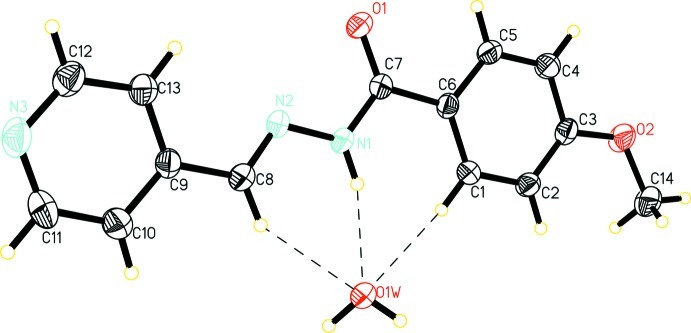
The molecular structure of (I) with displacement ellipsoids drawn at 30% probability level.

**Fig. 2.**
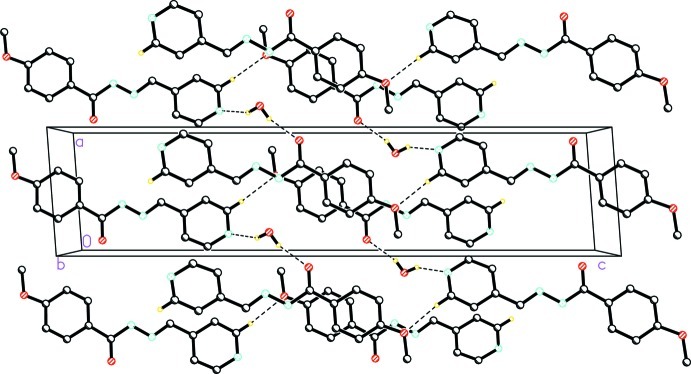
The crystal packing of the title compound I. Only hydrogen atoms involved in hydrogen bonding are shown.

## supplementary crystallographic information

### Comment

The diverse structural features and wide range of biological activities make Benzohydrazides as an importent class of organic compounds. The title compound is an structure analogue of Benzohydrazide, synthesize as a part of our ongoing research to study their varoius biological activities. The structure of title compound (Fig. 1) is similar to that of our recently published benzohydrazide derivative (*E*)-*N*'-(3,4-Dimethoxybenzylidene)-4-methoxybenzohydrazide (Taha *et al.*, 2012, Pv2573) with the difference that 3,4-dimethoxy phenyl ring is replaced by pyridine ring (N3/C9–C13). The azomethine (C=N,1.269 (3) Å) double bond adopt an *E* conformation (Fig. 1) with the torsion angle of 178.3 (19)° (N1–N2–C8–C9). Phenyl and pyridine rings (C1–C6 and N3/C9–C13) have a dihedral angle of 5.58 (12)° between them and maximum deviation of 0.006 (3) Å for C13 atoms from the root mean square plane. The bond lengths and angle were found to be similar as in structurally realted compounds (Fun *et al.*, 2011, Lu *et al.*, 2009, Zhang *et al.*, 2009). In the crystal structure molecules are consolidated by C11—H11A···O2 intermolecular hydrogen bonds (Fig.2) and extended to form a two-dimensional-network due to O1W—H1···O1 and O1W—H2···N3 (symmetry codes as in Table 2) intermolecular linkages made by water solvates (Fig. 2).

### Experimental

A mixture of 2 mmol of 4-methoxybenzohydrazide (0.332 g), 2 mmol isonicotinaldehyde (0.214 g) and catalytical amount of acetic acid was refluxed in methanol (20 ml) for 3 h. The progress of reaction was monitored by TLC. After completion of the reaction, the solvent was evaporated by vacuum to afford the crude product, which was dissolved and recrystallized from methanol to obtain colourless blocks (0.418 g in 82% yield).

### Refinement

H atoms on Methyl, phenyl, methine, nitrogen and water were positioned geometrically with C—H = 0.95 Å, CH_3_ = 0.93 Å, NH = 0.86 Å and O–H = 0.83–0.90 Å and constrained to ride on their parent atoms with *U*_iso_(H)= 1.5*U*_eq_(CH_3_, OH) and 1.2*U*_eq_(CH, NH). A rotating group model was applied to the methyl group.

### Crystal data

**Table:**

C_14_H_13_N_3_O_2_·H_2_O	*F*(000) = 576
*M**_r_* = 273.29	*D*_x_ = 1.321 Mg m^−^^3^
Monoclinic, *P*2_1_/*c*	Mo *K*α radiation, λ = 0.71073 Å
*a* = 6.6878 (5) Å	Cell parameters from 1112 reflections
*b* = 7.0420 (5) Å	θ = 2.8–22.8°
*c* = 29.249 (2) Å	µ = 0.10 mm^−^^1^
β = 94.233 (2)°	*T* = 273 K
*V* = 1373.74 (17) Å^3^	Block, colourless
*Z* = 4	0.20 × 0.17 × 0.10 mm

### Data collection

**Table:**

Bruker SMART APEX CCD diffractometer	2560 independent reflections
Radiation source: fine-focus sealed tube	1548 reflections with *I* > 2σ(*I*)
Graphite monochromator	*R*_int_ = 0.038
ω scan	θ_max_ = 25.5°, θ_min_ = 1.4°
Absorption correction: multi-scan (*SADABS*; Bruker, 2000)	*h* = −8→8
*T*_min_ = 0.981, *T*_max_ = 0.991	*k* = −7→8
7767 measured reflections	*l* = −35→35

### Refinement

**Table:**

Refinement on *F*^2^	Primary atom site location: structure-invariant direct methods
Least-squares matrix: full	Secondary atom site location: difference Fourier map
*R*[*F*^2^ > 2σ(*F*^2^)] = 0.047	Hydrogen site location: inferred from neighbouring sites
*wR*(*F*^2^) = 0.146	H-atom parameters constrained
*S* = 1.03	*w* = 1/[σ^2^(*F*_o_^2^) + (0.070*P*)^2^] where *P* = (*F*_o_^2^ + 2*F*_c_^2^)/3
2560 reflections	(Δ/σ)_max_ < 0.001
182 parameters	Δρ_max_ = 0.16 e Å^−^^3^
0 restraints	Δρ_min_ = −0.20 e Å^−^^3^

### Special details

**Table:**

Geometry. All e.s.d.'s (except the e.s.d. in the dihedral angle between two l.s. planes) are estimated using the full covariance matrix. The cell e.s.d.'s are taken into account individually in the estimation of e.s.d.'s in distances, angles and torsion angles; correlations between e.s.d.'s in cell parameters are only used when they are defined by crystal symmetry. An approximate (isotropic) treatment of cell e.s.d.'s is used for estimating e.s.d.'s involving l.s. planes.
Refinement. Refinement of *F*^2^ against ALL reflections. The weighted *R*-factor *wR* and goodness of fit *S* are based on *F*^2^, conventional *R*-factors *R* are based on *F*, with *F* set to zero for negative *F*^2^. The threshold expression of *F*^2^ > σ(*F*^2^) is used only for calculating *R*-factors(gt) *etc*. and is not relevant to the choice of reflections for refinement. *R*-factors based on *F*^2^ are statistically about twice as large as those based on *F*, and *R*- factors based on ALL data will be even larger.

### Fractional atomic coordinates and isotropic or equivalent isotropic displacement parameters (Å^2^)

**Table:**

	*x*	*y*	*z*	*U*_iso_*/*U*_eq_	
O1	0.0974 (2)	0.3345 (3)	0.05572 (5)	0.0607 (6)	
O2	0.6325 (2)	0.1818 (3)	−0.10876 (5)	0.0590 (5)	
N1	0.3945 (3)	0.3260 (3)	0.09758 (6)	0.0446 (5)	
H1A	0.5220	0.3083	0.0985	0.053*	
N2	0.3018 (3)	0.3646 (3)	0.13646 (6)	0.0436 (5)	
N3	0.1587 (4)	0.5223 (4)	0.29858 (7)	0.0662 (7)	
C1	0.5874 (3)	0.2320 (3)	0.01504 (7)	0.0413 (6)	
H1B	0.6640	0.2206	0.0428	0.050*	
C2	0.6754 (3)	0.1988 (3)	−0.02547 (7)	0.0424 (6)	
H2A	0.8102	0.1659	−0.0250	0.051*	
C3	0.5623 (3)	0.2146 (3)	−0.06665 (7)	0.0424 (6)	
C4	0.3619 (3)	0.2658 (4)	−0.06705 (8)	0.0513 (7)	
H4A	0.2858	0.2773	−0.0948	0.062*	
C5	0.2756 (3)	0.2993 (3)	−0.02691 (8)	0.0462 (6)	
H5A	0.1412	0.3340	−0.0277	0.055*	
C6	0.3867 (3)	0.2821 (3)	0.01516 (7)	0.0380 (5)	
C7	0.2800 (3)	0.3160 (3)	0.05702 (7)	0.0411 (6)	
C8	0.4126 (4)	0.3827 (4)	0.17334 (7)	0.0469 (6)	
H8A	0.5506	0.3663	0.1732	0.056*	
C9	0.3230 (3)	0.4292 (3)	0.21603 (7)	0.0429 (6)	
C10	0.4319 (4)	0.4088 (4)	0.25789 (8)	0.0544 (7)	
H10A	0.5630	0.3639	0.2592	0.065*	
C11	0.3438 (5)	0.4556 (4)	0.29754 (9)	0.0650 (8)	
H11A	0.4189	0.4395	0.3253	0.078*	
C12	0.0554 (4)	0.5413 (4)	0.25821 (9)	0.0580 (7)	
H12A	−0.0747	0.5880	0.2580	0.070*	
C13	0.1279 (4)	0.4965 (4)	0.21676 (8)	0.0499 (6)	
H13A	0.0476	0.5110	0.1896	0.060*	
C14	0.8422 (4)	0.1550 (5)	−0.11117 (9)	0.0640 (8)	
H14A	0.8706	0.1333	−0.1424	0.096*	
H14B	0.8852	0.0473	−0.0929	0.096*	
H14C	0.9124	0.2663	−0.0998	0.096*	
O1W	0.8218 (2)	0.2877 (3)	0.12291 (5)	0.0657 (6)	
H1	0.9224	0.2994	0.1078	0.098*	
H2	0.8700	0.2103	0.1461	0.098*	

### Atomic displacement parameters (Å^2^)

**Table:**

	*U*^11^	*U*^22^	*U*^33^	*U*^12^	*U*^13^	*U*^23^
O1	0.0361 (10)	0.1027 (16)	0.0440 (10)	0.0027 (10)	0.0085 (7)	0.0049 (9)
O2	0.0496 (10)	0.0914 (15)	0.0373 (9)	−0.0042 (10)	0.0115 (8)	−0.0133 (9)
N1	0.0347 (10)	0.0645 (15)	0.0357 (11)	0.0026 (10)	0.0104 (8)	0.0015 (9)
N2	0.0415 (11)	0.0552 (13)	0.0352 (11)	0.0018 (10)	0.0111 (9)	0.0016 (9)
N3	0.0749 (17)	0.0805 (18)	0.0450 (14)	−0.0011 (14)	0.0167 (12)	−0.0098 (12)
C1	0.0384 (13)	0.0493 (15)	0.0358 (12)	0.0007 (11)	0.0012 (10)	0.0032 (10)
C2	0.0360 (13)	0.0508 (16)	0.0410 (13)	0.0012 (11)	0.0057 (10)	−0.0028 (11)
C3	0.0419 (13)	0.0485 (15)	0.0378 (13)	−0.0085 (11)	0.0102 (10)	−0.0054 (11)
C4	0.0428 (14)	0.075 (2)	0.0359 (14)	−0.0049 (13)	−0.0009 (10)	−0.0017 (12)
C5	0.0340 (13)	0.0624 (18)	0.0424 (14)	−0.0034 (12)	0.0034 (10)	0.0007 (12)
C6	0.0378 (12)	0.0403 (14)	0.0366 (12)	−0.0053 (11)	0.0068 (9)	0.0025 (10)
C7	0.0370 (13)	0.0488 (16)	0.0379 (13)	−0.0027 (11)	0.0057 (10)	0.0049 (11)
C8	0.0394 (13)	0.0605 (17)	0.0416 (14)	0.0046 (12)	0.0084 (11)	0.0005 (12)
C9	0.0454 (14)	0.0469 (16)	0.0370 (13)	−0.0016 (12)	0.0074 (10)	0.0001 (11)
C10	0.0537 (15)	0.0636 (19)	0.0456 (15)	0.0042 (14)	0.0016 (11)	−0.0006 (13)
C11	0.081 (2)	0.077 (2)	0.0368 (15)	0.0004 (17)	0.0010 (13)	−0.0006 (13)
C12	0.0553 (16)	0.0642 (19)	0.0560 (17)	−0.0004 (14)	0.0137 (13)	−0.0108 (14)
C13	0.0504 (15)	0.0574 (17)	0.0423 (14)	0.0025 (13)	0.0059 (11)	−0.0048 (12)
C14	0.0565 (17)	0.087 (2)	0.0516 (16)	0.0062 (16)	0.0226 (13)	−0.0059 (14)
O1W	0.0371 (9)	0.1143 (17)	0.0465 (10)	0.0079 (10)	0.0095 (7)	0.0166 (10)

### Geometric parameters (Å, º)

**Table:**

O1—C7	1.226 (2)	C5—H5A	0.9300
O2—C3	1.370 (3)	C6—C7	1.481 (3)
O2—C14	1.422 (3)	C8—C9	1.462 (3)
N1—N2	1.362 (2)	C8—H8A	0.9300
N1—C7	1.365 (3)	C9—C10	1.385 (3)
N1—H1A	0.8600	C9—C13	1.390 (3)
N2—C8	1.269 (3)	C10—C11	1.379 (3)
N3—C11	1.326 (3)	C10—H10A	0.9300
N3—C12	1.330 (3)	C11—H11A	0.9300
C1—C2	1.382 (3)	C12—C13	1.375 (3)
C1—C6	1.388 (3)	C12—H12A	0.9300
C1—H1B	0.9300	C13—H13A	0.9300
C2—C3	1.379 (3)	C14—H14A	0.9600
C2—H2A	0.9300	C14—H14B	0.9600
C3—C4	1.387 (3)	C14—H14C	0.9600
C4—C5	1.367 (3)	O1W—H1	0.8361
C4—H4A	0.9300	O1W—H2	0.9098
C5—C6	1.395 (3)		
			
C3—O2—C14	118.14 (18)	N1—C7—C6	116.9 (2)
N2—N1—C7	118.33 (18)	N2—C8—C9	119.9 (2)
N2—N1—H1A	120.8	N2—C8—H8A	120.1
C7—N1—H1A	120.8	C9—C8—H8A	120.1
C8—N2—N1	117.12 (19)	C10—C9—C13	117.0 (2)
C11—N3—C12	116.1 (2)	C10—C9—C8	120.7 (2)
C2—C1—C6	121.2 (2)	C13—C9—C8	122.3 (2)
C2—C1—H1B	119.4	C11—C10—C9	119.3 (2)
C6—C1—H1B	119.4	C11—C10—H10A	120.3
C3—C2—C1	119.6 (2)	C9—C10—H10A	120.3
C3—C2—H2A	120.2	N3—C11—C10	124.1 (3)
C1—C2—H2A	120.2	N3—C11—H11A	117.9
O2—C3—C2	124.7 (2)	C10—C11—H11A	117.9
O2—C3—C4	115.6 (2)	N3—C12—C13	124.5 (3)
C2—C3—C4	119.7 (2)	N3—C12—H12A	117.8
C5—C4—C3	120.5 (2)	C13—C12—H12A	117.8
C5—C4—H4A	119.8	C12—C13—C9	119.0 (2)
C3—C4—H4A	119.8	C12—C13—H13A	120.5
C4—C5—C6	120.8 (2)	C9—C13—H13A	120.5
C4—C5—H5A	119.6	O2—C14—H14A	109.5
C6—C5—H5A	119.6	O2—C14—H14B	109.5
C1—C6—C5	118.2 (2)	H14A—C14—H14B	109.5
C1—C6—C7	124.6 (2)	O2—C14—H14C	109.5
C5—C6—C7	117.2 (2)	H14A—C14—H14C	109.5
O1—C7—N1	121.0 (2)	H14B—C14—H14C	109.5
O1—C7—C6	122.1 (2)	H1—O1W—H2	101.5
			
C7—N1—N2—C8	−176.5 (2)	C1—C6—C7—O1	−169.8 (2)
C6—C1—C2—C3	−0.3 (3)	C5—C6—C7—O1	9.2 (3)
C14—O2—C3—C2	−9.1 (3)	C1—C6—C7—N1	10.1 (3)
C14—O2—C3—C4	171.4 (2)	C5—C6—C7—N1	−170.9 (2)
C1—C2—C3—O2	−178.8 (2)	N1—N2—C8—C9	178.37 (19)
C1—C2—C3—C4	0.7 (4)	N2—C8—C9—C10	165.7 (2)
O2—C3—C4—C5	179.1 (2)	N2—C8—C9—C13	−14.8 (4)
C2—C3—C4—C5	−0.4 (4)	C13—C9—C10—C11	−0.2 (4)
C3—C4—C5—C6	−0.3 (4)	C8—C9—C10—C11	179.4 (2)
C2—C1—C6—C5	−0.4 (3)	C12—N3—C11—C10	0.8 (4)
C2—C1—C6—C7	178.6 (2)	C9—C10—C11—N3	−0.7 (4)
C4—C5—C6—C1	0.7 (4)	C11—N3—C12—C13	0.1 (4)
C4—C5—C6—C7	−178.4 (2)	N3—C12—C13—C9	−1.0 (4)
N2—N1—C7—O1	−2.6 (3)	C10—C9—C13—C12	1.0 (4)
N2—N1—C7—C6	177.42 (18)	C8—C9—C13—C12	−178.5 (2)

### Hydrogen-bond geometry (Å, º)

**Table:**

*D*—H···*A*	*D*—H	H···*A*	*D*···*A*	*D*—H···*A*
O1*W*—H1···O1^i^	0.84	2.00	2.811 (2)	162
O1*W*—H2···N3^ii^	0.91	2.11	2.956 (3)	154
N1—H1*A*···O1*W*	0.86	2.08	2.911 (2)	161
C1—H1*B*···O1*W*	0.93	2.54	3.440 (3)	162
C8—H8*A*···O1*W*	0.93	2.48	3.272 (3)	143
C11—H11*A*···O2^iii^	0.93	2.47	3.375 (3)	165

Symmetry codes: (i) *x*+1, *y*, *z*; (ii) −*x*+1, *y*−1/2, −*z*+1/2; (iii) *x*, −*y*+1/2, *z*+1/2.
